# Identifying recurrent stone formers with machine learning: A single‐centre observational study

**DOI:** 10.1002/bco2.70176

**Published:** 2026-03-06

**Authors:** Pedro Amado, Daniel G. Fuster, Matteo Bargagli, Dominik Obrist, Fiona Burkhard, Beat Roth, Francesco Clavica, Shaokai Zheng

**Affiliations:** ^1^ ARTORG Center for Biomedical Engineering Research University of Bern Bern Switzerland; ^2^ Department of Nephrology and Hypertension, Inselspital, Bern University Hospital University of Bern Bern Switzerland; ^3^ Department for Biomedical Research University of Bern Bern Switzerland; ^4^ Department of Urology Kantonsspital Aarau Aarau Switzerland; ^5^ Department of Urology, Inselspital Bern University Hospital Bern Switzerland; ^6^ Department of Diagnostic and Interventional Neuroradiology Bern University Hospital Bern Switzerland; ^7^ Department of Neurology, Inselspital Bern University Hospital Bern Switzerland

**Keywords:** classification, kidney stone, machine learning, recurrence

## Abstract

**Objectives:**

Kidney stones affect 12% of the population over their lifetime. Recurrent kidney stones lead to repeated interventions and excessive healthcare costs. Despite progress in imaging and metabolic evaluations, models to accurately identify patients at high risk are missing. In this study, we investigate whether machine learning methods can facilitate early identification of recurrent kidney stone formers.

**Patients and Methods:**

This observational study included data from the single‐centric Bern Kidney Stone Registry. Each participant had at least one stone episode. Different data imputation techniques, such as kernel density estimation (KDE) imputation, median imputation and *k*‐nearest neighbour (KNN) imputation, were evaluated in a logistic regression model. Feature selection with recursive feature elimination was applied. A fivefold cross‐validation was conducted using an 80/20 split. The classification criterion was recurrent kidney stone event.

**Results:**

A total of 706 patients (median age, 47, 71.2% male) were included, and 563 (79.7%) had recurrent stone events. The median imputation yielded the best‐performing models. A mean receiver operating characteristic curve area under the curve (AUC) of 0.71 ± 0.03 was achieved on the held‐out test set. Estimated glomerular filtration rate (OR = 0.45, 95% CI: 0.42–0.49), age at first stone episode (OR = 0.50, 95% CI: 0.46–0.56), oxalate (OR = 1.83, 95% CI: 1.43–2.23) and pH (OR = 1.74, 95% CI: 1.47–1.89) were among the most descriptive features.

**Conclusion:**

Routinely collected clinical and laboratory variables can be potentially exploited to identify recurrent stone formers, and our machine learning approach achieved better performance than previously reported work. With further validation on external datasets, our routine could support clinicians in designing dietary, medical or surveillance strategies, thereby reducing recurrence rates and improving long‐term outcomes for patients with stone‐forming conditions.

## 1 INTRODUCTION

Kidney stone disease is a prevalent condition, affecting roughly 12% of the population over a lifetime, with peak incidence in adults aged 20 to 49 years old.^1^ The prevalence varies by geographic region, influenced by factors such as climate, diet and genetic predisposition.^2^ Worldwide, the incidence and recurrence rates are increasing,^3^ with recurrence being common in approximately 30–50% within 5–10 years and 75% within 20 years after the first stone event.^3^, ^4^ Recurrent stone formers face increased risks of pain, repeated surgeries and long‐term kidney damage, leading to substantial morbidity and healthcare costs.^5^, ^6^, ^7^ Accurate identification of patients at high risk of recurrent stone events (RSEs) is therefore essential.

Younger age at first stone, being a male, a positive family history and a personal history of previous stones are all associated with increased risk of RSE.^2^, ^8^, ^9^, ^10^ Certain stone types, such as cystine and infection‐related struvite stones, are particularly prone to recur unless their cause is effectively treated.^8^, ^11^ Additionally, metabolic abnormalities like hypercalciuria, hypocitraturia and hyperoxaluria,^12^, ^13^, ^14^, ^15^ as well as comorbidities such as obesity, gout, bowel disease, diabetes and smoking^12^, ^16^ contribute significantly to future risk of RSE. Genetic polymorphisms^17^ and diet seem to be important estimators too.^4^, ^9^ These factors are commonly assessed through patient history, stone analysis and metabolic evaluation, and they form the basis for clinical risk stratification.

Current clinical guidelines from the European Association of Urology^18^ recommend identifying high‐risk stone formers to guide preventive interventions such as dietary modifications or pharmacological therapy. Dietary interventions are often enough for first‐time stone formers, while pharmacological treatments in addition to dietary interventions are more frequently started in patients with RSE.^9^ Despite these guidelines, classifying recurrence remains challenging.^19^ Most tools classify patients using broad categories and do not provide individualized risk stratification. The ROKS (Risk of Recurrence of Kidney Stone) score is one of the few established models, using basic demographic and clinical data to estimate 5‐ or 10‐year recurrence risk.^20^ However, its predictive performance is moderate,^20^, ^21^, ^22^ and it does not include important clinical variables such as 24‐h urine metabolic parameters,^20^ which have been associated with recurrence risk.^15^, ^23^, ^24^

Machine learning (ML) offers a promising alternative to traditional categorical risk‐scoring systems, as ML models can integrate a wide range of features, including demographics, stone history, laboratory data and comorbidities, and provide personalized suggestions. Early applications of ML in nephrolithiasis have shown encouraging results, with models using clinical and urine chemistry data achieving reasonable predictive accuracy on recurrent RSE.^24^, ^25^, ^26^, ^27^

In this study, we developed and validated an ML pipeline to classify RSE in adult patients. The goal was to distinguish between individuals with only one stone and those with RSE using routinely collected clinical and biochemical data. Following the TRIPOD+AI guideline,^28^ multiple imputation strategies and feature selection techniques were evaluated using a fully nested cross‐validation scheme. The resulting classification model was designed to identify clinical and biochemical variables associated with RSE. This study offers insights into potential risk and protective factors, enhancing the understanding of RSE to inform targeted preventive strategies in clinical practice.

## 2 PATIENTS AND METHODS

### 2.1 Participants

This study used data from the Bern Kidney Stone Registry (BKSR), a retrospective registry established at the Division of Nephrology and Hypertension, Bern University Hospital, Switzerland. Data acquisition occurred between March 2004 and March 2020. The registry enrolled adults (≥18 years) with a history of at least one kidney stone event. Detailed inclusion criteria have been previously reported.^29^, ^30^, ^31^, ^32^, ^33^ All participants received standard‐of‐care management according to clinical guidelines. The study was conducted in accordance with the Declaration of Helsinki, and ethical approval was granted by the Ethical Committee of the Canton of Bern (KEK‐Bern 95/06). All participants provided written informed consent.

### 2.2 Classification criterion

The classification criterion was RSE, defined as more than one documented kidney stone event. Classification was reviewed by nephrologists based on clinical history and medical records, with patients categorized as either recurrent (≥2 episodes) or first‐time (1 episode) stone formers.

### 2.3 Data processing

Candidate features for each subject were selected based on clinical relevance and availability at the time of enrolment. These included demographic variables (e.g., age and sex), clinical characteristics (e.g., body mass index [BMI]) and biochemical measurements obtained during the baseline metabolic evaluation, such as 24‐h urinary parameters. Features with more than 25% missing data were excluded for model development. Rates of missing data are summarized in the Supporting Information (Table S1).

### 2.4 Data imputation

Missing values were addressed using three imputation methods: Kernel density estimation (KDE) for continuous features and Bernoulli probability distribution for binary data, respectively; median imputation for continuous features and random sampling (50/50) for binary data; and *k*‐nearest neighbour (KNN) for both feature types (see Supporting Information). All imputation methods were applied in a leakage‐free manner, where imputation parameters were derived from the training set and applied independently to the corresponding left‐out validation set. After imputation, continuous features were standardized using *z* score normalization (between −1 and 1), and features with pairwise Pearson correlation above 0.9 (based on the training data) were removed to reduce multicollinearity.

### 2.5 Model development

**FIGURE 1:**
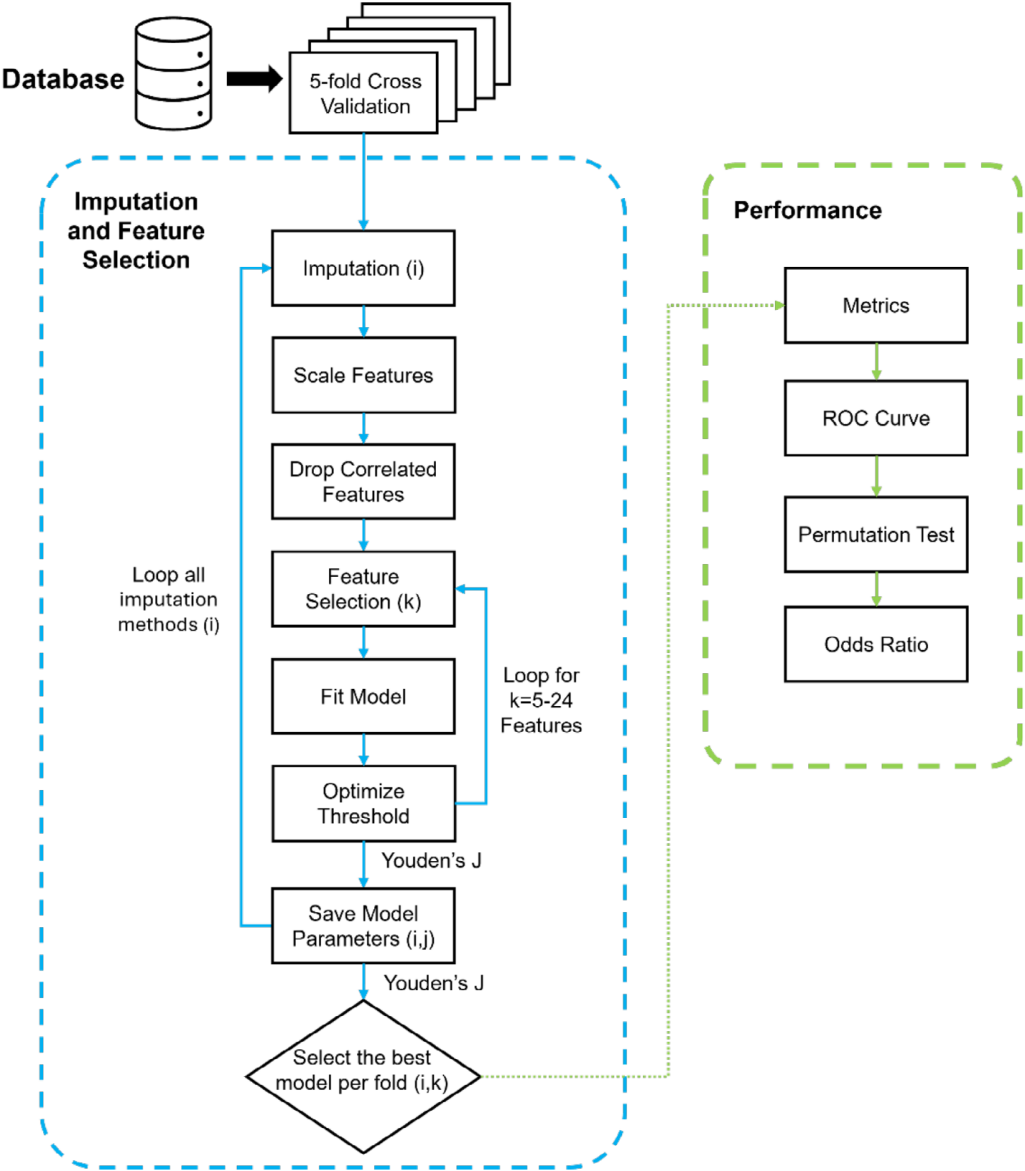
Flow chart of the modelling pipeline including imputation and feature selection, followed by performance evaluation.

A fivefold cross‐validation scheme was performed on the full dataset. Within each fold, all combinations of the three imputation methods and varying numbers of selected features (ranging from 5 to 24) were evaluated using logistic regression (scikit‐learn v 1.6.1). Feature selection began at five features, as fewer numbers of features showed poorer predictive performance in our preliminary studies. Feature selection was carried out using recursive feature elimination (RFE), as implemented in scikit‐learn (v 1.6.1), and logistic regression with balanced class weights was trained with a maximum of 3000 iterations. Classifier probabilities were generated, and the optimal binary decision threshold was determined by maximizing Youden's *J* statistics. The combination of the imputation method and the number of features that achieved the highest Youden's *J* in each training fold was selected as the best‐performing model for that fold. The full pipeline is shown in Figure 1.

### 2.6 Model validation

Using the five best models selected from each fold, performance was reported on both the training and the left‐out validation sets. Metrics included accuracy, recall, specificity, F1 score, balanced accuracy and receiver operating characteristic curve (ROC) area under the curve (AUC). To support model interpretability, odds ratios (ORs) were calculated for features consistently selected across all models. Features with OR > 1 are associated with an increased risk of RSE, whereas those with OR < 1 are considered protective.

### 2.7 Statistics

Results are reported in mean ± SD (standard deviation) or median (interquartile range, IQR) where appropriate. OR was reported with a 95% confidence interval (CI). Univariate comparisons were tested using the chi‐squared test for binary variables and the two‐sided Wilcoxon rank‐sum test (SciPy, 1.11.1) for continuous variables. *p* values smaller than 0.05 were regarded as significant.

A permutation test was performed on the best and worst performing cross‐validation models to assess whether the observed predictive performance was significantly better than expected by chance. Under the null hypothesis of no association between features and the classification criterion, the training labels were randomly permuted while preserving the validation set. For each permutation (*n* = 1000), the logistic regression model was retrained using the fixed parameters identified in the cross‐validation, and the ROC‐AUC was computed on the held‐out validation set. The *p* value was estimated as the proportion of permuted AUCs greater than or equal to the observed AUC.^34^

## 3 RESULTS

### 3.1 Patients

The overall cohort included *n* = 706 patients with a median age of 47 years (IQR: 36–58), and 503/706 (71.2%) were male. Characteristics for patients with and without recurrence are summarized in Table 1.

**TABLE 1:** Patient characteristics for the nonrecurrent and recurrent groups. *p* values between the two patient groups are reported. *p* values smaller than 0.05 are regarded as significant and highlighted in bold.

Category	Variable	Nonrecurrent	Recurrent	*p* value
Demographics	Age, year, median (IQR)	38 (27–53)	48 (39–59)	**<0.001**
	Sex (*n*, %)			**0.032**
	Female	52, 36.4%	151, 26.8%	
	Male	91, 63.6%	412, 73.2%	
Clinical examination	Body mass index, kg/m^2^, median (IQR)	25.6 (21.3–8.7)	26.4 (23.8–29.7)	**0.007**
	Systolic blood pressure, mmHg, median (IQR)	126.0 (117–0‐139.4)	133.0 (123.0–146.0)	**0.004**
	Diastolic blood pressure, mmHg median (IQR)	81.2 (74.0–92.0)	86.0 (78.0–95.0)	**0.005**
Medical history	Diabetes (*n*, %)			1.000
	Yes	4, 2.8%	15, 2.7%	
	No	139, 97.2%	547, 97.3%	
	Hypertension (*n*, %)			0.232
	Yes	43, 30.9%	192, 36.9%	
	No	96, 69.1%	329, 63.1%	
	Family history stones (*n*, %)			0.989
	Yes	56, 42.4%	232, 43.0%	
	No	76, 57.6%	308, 57.0%	
Stone history	Age 1^st^ stone, year, median (IQR)	36 (25–51)	34 (25–45)	0.102
Stone risk ratios	Supersaturation calcium oxalate, median (IQR)	6.0 (3.6–10.1)	6.8 (4.0–11.0)	0.145
	Supersaturation brushite, median (IQR)	1.0 (0.4–2.9)	1.2 (0.4–3.4)	0.500
	Supersaturation uric acid, median (IQR)	3.0 (0.9–6.2)	2.7 (0.6–7.1)	0.246
	Urine titratable acidity, median (IQR)	20.2 (10.5–30.6)	19.2 (9.9–28.2)	0.178
Laboratory urine 24 h	Total urine volume, litres, median (IQR)	1.9 (1.4–2.5)	2.0 (1.5–2.6)	0.116
	Urine pH, median (IQR)	5.8 (5.3–6.5)	5.9 (5.4–6.6)	0.124
	Components, mmol, median (IQR)			
	Sodium	171.0 (139.2–233.5)	179.5 (139.5–231.0)	0.524
	Potassium	62.0 (49.5–79.2)	61.5 (48.5–78.8)	0.712
	Uric acid	7.9 (5.9–10.0)	8.2 (6.2–10.1)	0.381
	Creatinine	13.0 (10.5–16)	13.4 (10.4–16.3)	0.575
	Calcium	5.4 (3.8–7.3)	5.6 (3.6–8.4)	0.297
	Magnesium	3.1 (3.1–5.1)	4.0 (3.0–5.2)	0.731
	Chloride	155.2 (124.5–233.1)	168.2 (126.6–216.0)	0.661
	Citrate	2.7 (1.8–3.5)	2.6 (1.7–3.6)	0.794
	Inorganic phosphate	29.5 (22.5–35.4)	28.3 (22.1–36.3)	0.963
	Oxalate	0.4 (0.2–0.5)	0.4 (0.4–0.6)	**0.013**
	Sulfate	20.0 (16.2–26.4)	20.9 ()	0.917
	eGFR, median (IQR)	100.3 (86.6–117.6)	95.3 (79.1–108.3)	**<0.001**
Stone composition	Total proportion of calcium oxalate stones (%)	100 (75–100)	95 (65–100)	0.495
	Proportion of calcium oxalate monohydrate	70 (10–80)	70 (10–80)	0.720
	Proportion of calcium oxalate dihydrate	20 (5–30)	20 (0–30)	0.492
	Total proportion of calcium phosphate stones (%)	0 (0–10)	0 (0–10)	0.799
	Proportion of uric acid	0 (0–0)	0 (0–0)	0.689
	Proportion of struvite	0 (0–0)	0 (0–0)	0.939
	Proportion of cystine	0 (0–0)	0 (0–0)	0.896
	Total proportion of other stone groups (%)	0 (0–0)	0 (0–0)	0.966

Univariate analysis comparing both patient groups, performed on all 35 features, showed that RSE was associated with higher age (*p* < 0.001), higher systolic and diastolic blood pressure (*p* < 0.01) and a higher BMI (*p* < 0.01). Urinary biochemical profiles and stone composition variables were largely similar between the two groups, except for urinary oxalate (*p* < 0.05) and estimated glomerular filtration rate (eGFR) (*p* < 0.001). Patients with recurrence had higher urinary oxalate excretion and lower eGFR compared with those without recurrence. No significant differences were observed regarding diabetes, hypertension or family history of stones.

Moreover, 24‐h urine chemistry was used to calculate the relative supersaturation of uric acid, calcium oxalate and brushite, providing an estimate of the risk of crystal formation. However, no statistically significant difference was observed between the recurrence and nonrecurrence groups. These metrics are obtained by comparing the ionic activity product of each salt to its thermodynamic solubility product.^35^, ^36^ Required inputs include urinary pH, volume, calcium, oxalate, uric acid, citrate and other electrolytes.

### 3.2 Imputation methods

A total of 35 features were included, and 11 of them had more than 25% missing data. Twenty‐four features were considered for subsequent analysis. Further details are reported in the Supporting Information (Table S1).

When evaluating the different imputation methods, the KDE method preserved the original distribution across all features. In contrast, the KNN method maintained similarity for simpler features but introduced moderate shifts in more complex or skewed features. The median method consistently altered feature distributions by introducing a peak at the median value, reducing overall variability. Examples for each method are given in the Supporting Information (Figure S1).

**FIGURE 2:**
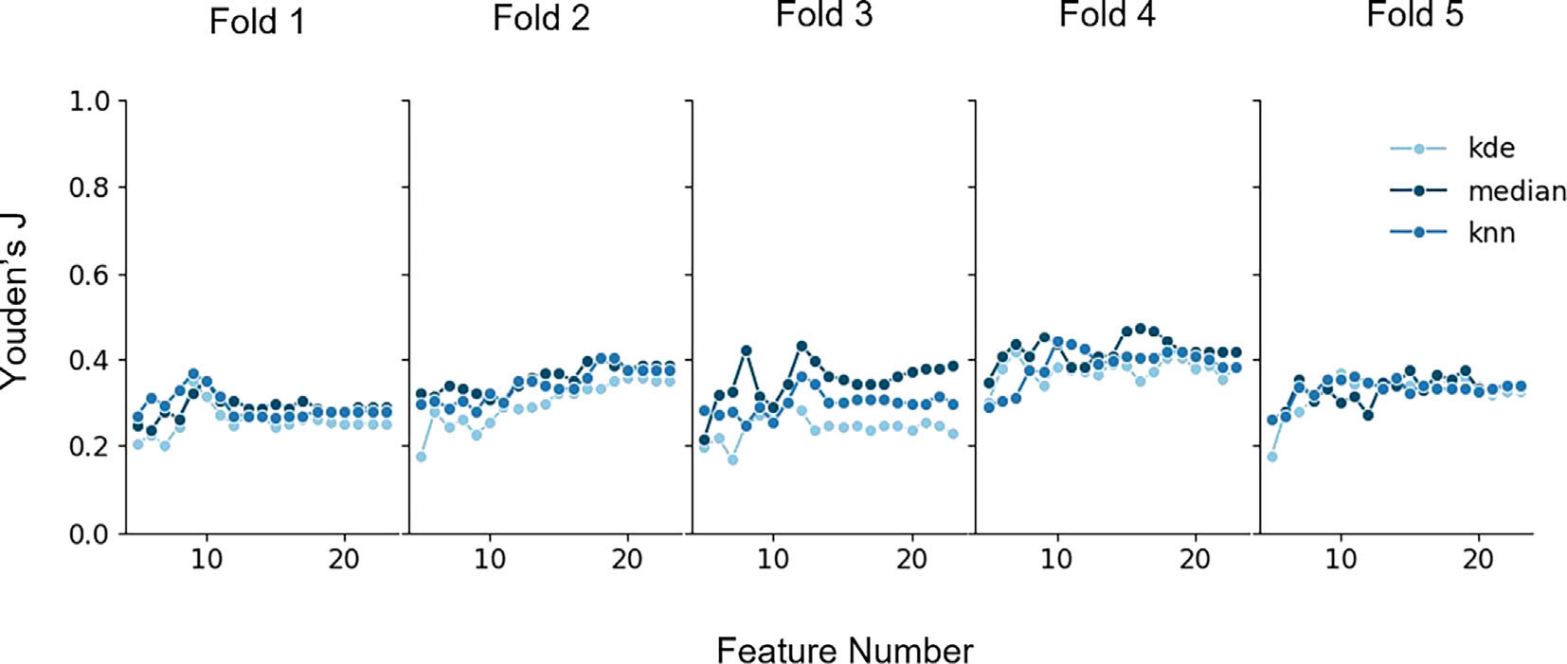
Imputation method and feature selection. Each panel shows the mean Youden's J for the three selected imputation methods (KDE, Median, and KNN) and for feature count varying from five to 25 features.

During model development, combinations of the three imputation methods and varying numbers of selected features were evaluated. Figure 2 summarizes the performance of each configuration across all fivefold cross‐validation. In four out of fivefolds, median imputation obtained the highest Youden's *J* index, with optimal feature counts ranging from 12 to 18. In the remaining fold, KNN imputation achieved the best performance with nine selected features.

### 3.3 Model specification and performance

**FIGURE 3:**
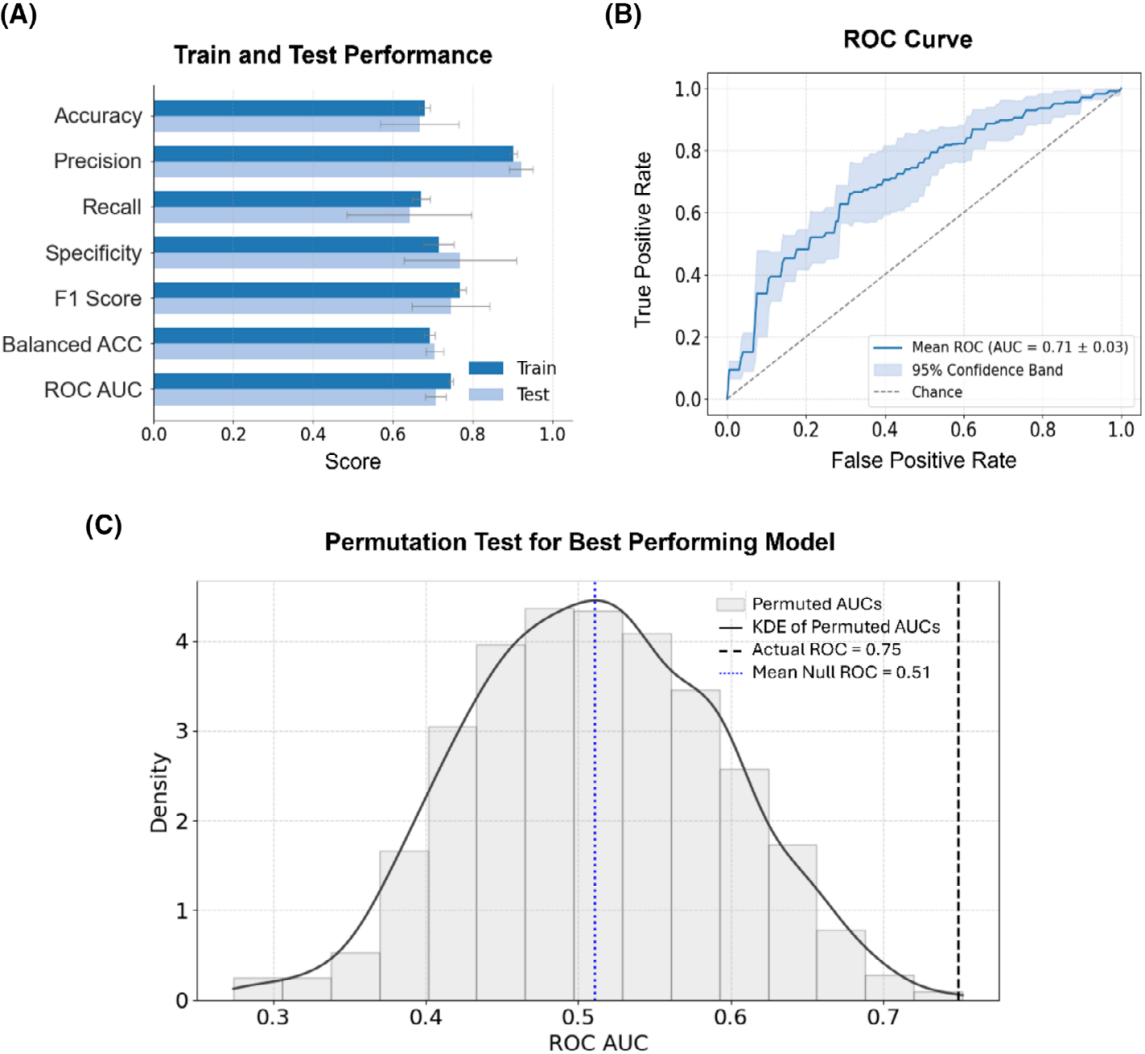
Performance metrics and ROC curve. (A) Train (dark blue) and test (light blue) performance metrics. (B) Mean and 95% confidence interval for ROC across the fivefolds on the validation set. (C) Permutation test for the best performing model out of the fivefolds.

The model's performance on the fivefold cross‐validation is shown in Figure 3A. For the training and testing data, the mean AUC was 0.74 ± 0.01 and 0.71 ± 0.02, the F1 score was 0.77 ± 0.01 and 0.71 ± 0.10, the balanced accuracy was 0.74 ± 0.01 and 0.70 ± 0.02, the accuracy was 0.68 ± 0.01 and 0.62 ± 0.10, the recall was 0.67 ± 0.02 and 0.58 ± 0.16, the precision was 0.90 ± 0.01 and 0.92 ± 0.03 and the specificity was 0.71 ± 0.04 and 0.79 ± 0.14.

The best‐performing model across the five cross‐validation folds achieved an AUC of 0.75 (Figure 3B). For the permutation test, the best‐performing fold (AUC = 0.749, median imputation method) had a mean AUC of 0.511 ± 0.083 (Figure 3C). The corresponding *p* value of 0.002 indicates that the observed predictive performance would be highly unlikely if there were no association between the features and the classification criterion. For the worst‐performing fold (AUC = 0.680, KNN imputation), the mean AUC was 0.505 ± 0.095 (Figure S2), with a *p* value of 0.025, still supporting a performance significantly above chance.

**FIGURE 4:**
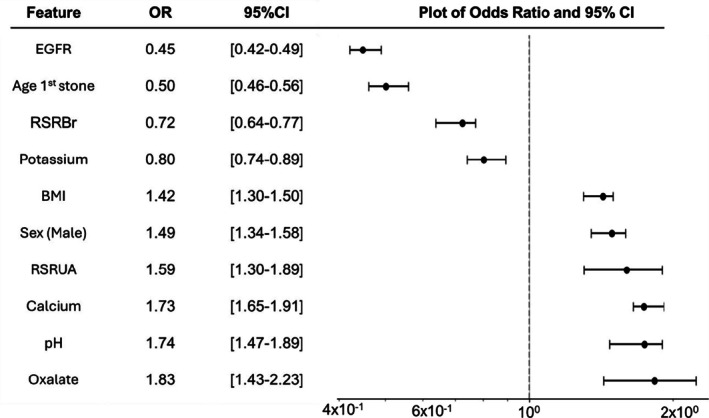
Summary of key features. Odds ratios with confidence intervals from the final logistic regression model.

The summary of the most influential features selected by the models is presented in Figure 4. Only features selected by at least four out of the fivefold models were included. Notably, a higher urine pH (OR = 1.74, 95% CI: 1.47–1.89), elevated calcium (OR = 1.73, 95% CI: 1.65–1.91) and oxalate (OR = 1.83, 95% CI: 1.43–2.23) concentrations and increased supersaturation of uric acid (OR = 1.59, 95% CI: 1.30–1.89) were consistently associated with a greater risk of RSE. Other risk factors with OR > 1 include male sex (OR = 1.49, 95% CI: 1.34–1.58) and higher BMI (OR = 1.42, 95% CI: 1.30–1.50), both of which appear to increase the likelihood of recurrent stone events.

Features associated with a reduced risk of recurrence included higher urinary potassium (OR = 0.80, 95% CI: 0.74–0.89) and supersaturation of brushite (RSRBr, OR = 0.72, 95% CI: 0.64–0.77). The most protective features identified were eGFR (OR = 0.72, 95% CI: 0.64–0.77) and age at first stone episode (OR = 0.50, 95% CI: 0.46–0.56).

## 4 DISCUSSION

In this study, we developed and validated a fully nested ML pipeline to classify patients with RSE versus those who had experienced only a single documented episode, based on routinely available demographic and 24‐h urine laboratory data. The classifier achieved strong performance, with a mean ROC‐AUC of 0.71 ± 0.03. Statistical analysis between the different available features was also evaluated. All variables that showed statistically significant differences between recurrent and nonrecurrent groups in the univariate analysis (*p* < 0.05) in Table 1, such as BMI, sex, eGFR and oxalate, were also selected by the classification model. Interestingly, some variables that did not show significant differences in univariate statistical tests still emerged as important features in the logistic regression model. This highlights the added value of multivariable modelling in uncovering complex relationships that may not be evident through univariate analysis.

Our findings complement and extend prior work using applied statistical and ML methods to estimate RSE. For example, the ROKS nomogram developed by Rule et al.,^20^ achieved a C‐index of approximately 0.67 in internal validation and an AUC of 0.66 in external surgical cohorts, using 11 features. More recent ML approaches,^24^, ^25^ incorporating 24‐h urine data and electronic health records, reported AUC between 0.60 and 0.65. While these studies demonstrate the feasibility of ML in this domain, their improvements over traditional models were modest. In contrast, our classification model demonstrated good performance between the target groups. This suggests that careful integration of clinical and biochemical features, combined with ML models, could substantially improve the success rate of identifying patients with RSE. If trained and validated on a larger external dataset, the pipeline presented in this study may facilitate a more accurate risk stratification tool for clinical practice.

Handling missing data is critical in developing classification models, particularly in medicine, where missingness can vary substantially. Among the three tested imputation strategies (KDE, KNN, median), median imputation was most often selected across cross‐validation folds, indicating the most consistent performance in our setting. This is likely due to the lower variance in the data, which makes it well‐suited to our dataset, given its relatively low levels of missing data (<6.5%). The KNN method, though less frequently chosen, still showed competitive performance by leveraging similarities between patients, consistent with reports that multivariate approaches can improve classification in structured clinical data.^37^, ^38^, ^39^, ^40^ By contrast, the KDE method performed worse for the classification task explored in this study. This may be because it samples from the overall distribution of a variable, maintaining the variability of the original data. While this makes KDE more faithful to the underlying data distribution, the variances are substantially larger in the imputed data compared with the other two methods. As such, the KDE method might offer better descriptive values in generative models intended for a larger cohort, subject to further examination.

One of the main advantages of using logistic regression in clinical classification is its interpretability, since it can quantify how individual features influence the odds of outcomes such as RSE. In our model, several features demonstrated strong and clinically meaningful associations. The degree of variability in these estimates reflects the stability of each feature's influence across different models. Although the optimal number of features varied slightly across models, a core subset of features was repeatedly selected, and models with more than 10 features consistently demonstrated strong discriminative ability. Features count below nine, however, were associated with reduced performance.

The eGFR was the strongest protective factor in our model. Higher eGFR values, associated with better kidney function, were linked to a lower risk of recurrence. This supports the idea that healthy kidneys are more effective at clearing stone‐forming substances, thereby reducing the likelihood of new stones.^41^ Age at first stone episode was also a protective factor, showing its inverse correlation with RSE. Younger age at first presentation often indicates a more aggressive disease, which is associated with a higher lifetime recurrence risk.^42^ Potassium levels also showed protective effects, aligning with established evidence that hypokalaemia is associated with a higher risk of kidney stones.^43^ This is explained by the role of potassium in maintaining urinary citrate levels, a key inhibitor of calcium stone formation.^43^

Conversely, several features were associated with increased recurrence risk. BMI was positively associated with recurrence, reinforcing the well‐established connection between obesity and stone formation.^44^ Male sex is also classified as a risk factor for recurrence odds, reflecting patterns possibly related to dietary, hormonal or urinary compositional differences.^45^ Urinary markers in our data reflected known risk factors for stone formation, especially calcium oxalate stones, which were the most common type in our cohort. Higher urinary calcium was linked to increased recurrence risk, consistent with the role of excess calcium in crystal formation.^8^ Urinary pH also played a role. Low pH can lead to uric acid stones, while high pH can favour calcium phosphate stones, suggesting that both very low and very high pH levels may increase risk, depending on the stone type.^46^ Although pH mainly affects other stone types, our data suggest that higher urinary pH may also increase the risk of calcium oxalate stone recurrence, possibly due to mixed stone formation or metabolic factors like low citrate or high urinary calcium. Moreover, urinary oxalate showed the strongest association with RSE overall. Since oxalate binds easily with calcium to form calcium oxalate, which is the main stone type in our patients, even small increases in oxalate levels can raise the risk, especially when urine volume is low or when inhibitors like citrate are reduced.

Interestingly, the relative supersaturation values also emerged as key features. Typically, these metrics are not used for classifying recurrence risk directly, but rather to assess stone‐forming potential based on a 24‐h urine laboratory test.^35^, ^47^ Clinically, they guide preventive strategies by quantifying the thermodynamic likelihood of crystal formation, informing dietary and pharmacologic interventions. Their importance in our model suggests that the effects that lead to stone formation help classify RSE. This supports the clinical value of relative supersaturation indices for both treatment decisions and classification.

Overall, these findings corroborate established mechanisms of stone formation while providing information on the critical features that lead to RSE. Importantly, many of the identified risk factors can be adjusted through dietary, pharmacological or lifestyle interventions, which can be used as prevention strategies.

Several limitations of our study should be noted. First, the dataset was derived from a cross‐sectional cohort where recurrence was defined independently of prior or subsequent treatment. As such, the present analysis cannot assess the impact of treatment on recurrence risk. Future longitudinal studies are therefore needed to determine whether these predictors can identify patients who remain at elevated risk of recurrence despite treatment. Furthermore, we acknowledge that the study was conducted using data from a single centre and without external validation, which may limit generalizability. While nested cross‐validation provides an unbiased internal estimation of model performance, future work should focus on multicentre external validation and prospective data collection, including information on time to recurrence and number of recurrence events, to further refine risk assessment and clinical applicability. Nonetheless, given the strong performance achieved by using data from a single centre and only routinely available variables, our approach presents itself as a viable candidate for further development to help personalize preventive strategies for stone formers with a high risk of RSE, who would otherwise be missed.

Furthermore, although the model achieved a high AUC, suggesting good overall performance, its lower recall compared with the AUC indicates that some recurrent cases were not identified. Clinically, this means the tool may underestimate recurrence risk in certain patients. Future work should aim to improve sensitivity while maintaining discrimination. Moreover, our sample size was relatively small (*n* = 706), with an even smaller number of nonrecurrent cases (*n* = 143). Recurrence was not stratified by time to recurrence, which limited the temporal interpretation of the data. In contrast, other studies, such as Doyle et al.,^25^ used symptom‐based definitions, and the ROKS nomogram stratified recurrence risk at multiple time points.

## AUTHOR CONTRIBUTIONS

Pedro Amado and Shaokai Zheng contributed to the conception and design of the study, performed the data analysis and wrote the early drafts of the manuscript. Francesco Clavica acquired funding for this study. Daniel G. Fuster and Beat Roth collected the data. Matteo Bargagli and Pedro Amado contributed to data curation. All authors provided critical reviews of the manuscript.

## CONFLICT OF INTEREST STATEMENT

The authors declare that the research was conducted in the absence of any commercial or financial relationships that could be construed as a potential conflict of interest.

## Supporting information


**Table S1.** **Patient characteristics and missing data.** Summary of patient information for the full dataset, including the percentage of missing values for each variable.
**Figure S1. Distribution of imputed data across methods.** Distributions of age of 1st stone, RSRCaOx, and hypertension before and after imputation using KDE, median, and KNN methods. Plots illustrate how each method affects data distribution.
**Figure S2.** Permutation test for the worst performing model out of the 5 folds.

## Data Availability

The data that support the findings of this study are available from the corresponding author upon reasonable request.
