# Supplementary material for: Identifying recurrent stone formers with machine learning: A single‐centre observational study
Source: BJUI Compass. 2026 Mar 6;7(3):e70176. doi: 10.1002/bco2.70176 (PMC12966608; doi:10.1002/bco2.70176)
Supplement: Supplementary file 1 — Table S1. Patient characteristics and missing data. Summary of patient information for the full dataset, including the percentage of missing values for each variable. Figure S1. Distribution of imputed data across methods. Distributions of age of 1st stone, RSRCaOx, and hypertension before and after imputation using KDE, median, and KNN methods. Plots illustrate how each method affects data distribution. Figure S2. Permutation test for the worst performing model out of the 5 folds. [file BCO2-7-e70176-s001.docx]

**Supplementary material**

**Data imputation**

The kernel density estimation (KDE) imputation was used to estimate the underlying probability distribution of observed values using a Gaussian kernel, with bandwidth selected via Scott’s rule [52]. Missing values were then filled by random sampling from this estimated distribution. For binary variables, missing values were imputed using Bernoulli sampling based on the observed probability of the positive class.

The median imputation involved replacing missing values in continuous variables with the median of the available data. Binary variables under this strategy were imputed by drawing random values (0 or 1) with equal probability.

The k-nearest neighbor (KNN) imputation used the five most similar observations (based on Euclidean distance) to estimate missing values. For binary variables, the resulting imputed values were then thresholded at 0.5 to restore binary format (values ≥ 0.5 were set to 1, others to 0).

**Table S1 –** **Patient characteristics and missing data.** Summary of patient information for the full dataset, including the percentage of missing values for each variable.

| Category | Variable | Summary | Missing (n, %) |
| --- | --- | --- | --- |
| *Demographics* | Age, yr, median (IQR) | 47 (36-58) | 0 (0.0%) |
|  | Sex (n, %) |  | 0 (0.0%) |
|  | Male | 503 (71.2%) |  |
|  | Female | 203 (28.8%) |  |
| *Clinical Examination* | Body mass index, kg/m^2^, median (IQR) | 26.1 (23.4-29.3) | 27 (3.8%) |
|  | Systolic blood pressure, mmHg, median (IQR) | 132 (122-146) | 233 (33.0%) |
|  | Diastolic blood pressure, mmHg median (IQR) | 85 (78-95) | 233 (33.0%) |
| *Medical History* | Diabetes (n, %) |  | 1 (0.1%) |
|  | Yes | 19 (2.7%) |  |
|  | No | 686 (97.2%) |  |
|  | Hypertension (n, %) |  | 46 (6.5%) |
|  | Yes | 235 (33.3%) |  |
|  | No | 425 (60.2%) |  |
|  | Family history stones (n, %) |  | 34 (4.8%) |
|  | Yes | 288 (40.8%) |  |
|  | No | 384 (54.4%) |  |
| *Stone History* | Total stones, No, median (IQR) | 3 (2-4) | 0 (0.0%) |
|  | Age 1^st^ stone, yr, median (IQR) | 35 (25-47) | 43 (6.1%) |
|  | Previous stone events (n, %) |  | 0 (0.0%) |
|  | Yes | 563 (79.7%) |  |
|  | No | 143 (20.3%) |  |
| *Stone Risk Ratios* | Supersaturation Calcium Oxalate, median (IQR) | 6.7 (4.0-10.6) | 43 (6.1%) |
|  | Supersaturation Brushite, median (IQR) | 1.2 (0.4-3.4) | 32 (4.5%) |
|  | Supersaturation Uric Acid, median (IQR) | 2.8 (0.6-7.0) | 30 (4.2%) |
|  | Urine titratable acidity, median (IQR) | 19.4 (10.1-28.6) | 23 (3.3%) |
| *Laboratory Urine 24h* | Total urine volume, liters, median (IQR) | 2.0 (1.5-2.6) | 7 (1.0%) |
|  | Urine pH, median (IQR) | 5.9 (5.4-6.6) | 20 (2.8%) |
|  | Components, mmol, median (IQR) |  |  |
|  | Sodium | 177.3 (139.5-232.3) | 14 (2.0%) |
|  | Potassium | 61.8 (48.5-79.0) | 16 (2.3%) |
|  | Uric acid | 8.1 (6.1-10.1) | 14 (2.0%) |
|  | Creatinine | 13.3 (10.5-16.3) | 11 (1.6%) |
|  | Calcium | 5.6 (3.6-8.1) | 15 (2.1%) |
|  | Magnesium | 4.0 (3.0-5.2) | 22 (3.1%) |
|  | Chloride | 166.0 (124.9-217.1) | 18 (2.5%) |
|  | Citrate | 2.6 (1.7-3.5) | 21 (3.0%) |
|  | Inorganic phosphate | 28.7 (22.2-36.1) | 16 (2.3%) |
|  | Oxalate | 0.4 (0.3-0.6) | 21 (3.0%) |
|  | Sulfate | 20.9 (15.2-26.7) | 21 (3.0%) |
|  | eGFR, median (IQR) | 96.5 (80.3-109.5) | 7 (1.0%) |
| *Stone Composition* | Total proportion of calcium oxalate stones (%) | 100 (70-100) | 177 (25.1%) |
|  | Proportion of calcium oxalate monohydrate | 70 (10-80) | 181 (25.6%) |
|  | Proportion of calcium oxalate dihydrate | 20 (0-30) | 181 (25.6%) |
|  | Total proportion of calcium phosphate stones (%) | 0 (0-10) | 177 (25.1%) |
|  | Proportion of uric acid | 0 (0-0) | 177 (25.1%) |
|  | Proportion of struvite | 0 (0-0) | 177 (25.1%) |
|  | Proportion of cystine | 0 (0-0) | 177 (25.1%) |
|  | Total proportion of other stone groups (%) | 0 (0-0) | 177 (25.1%) |


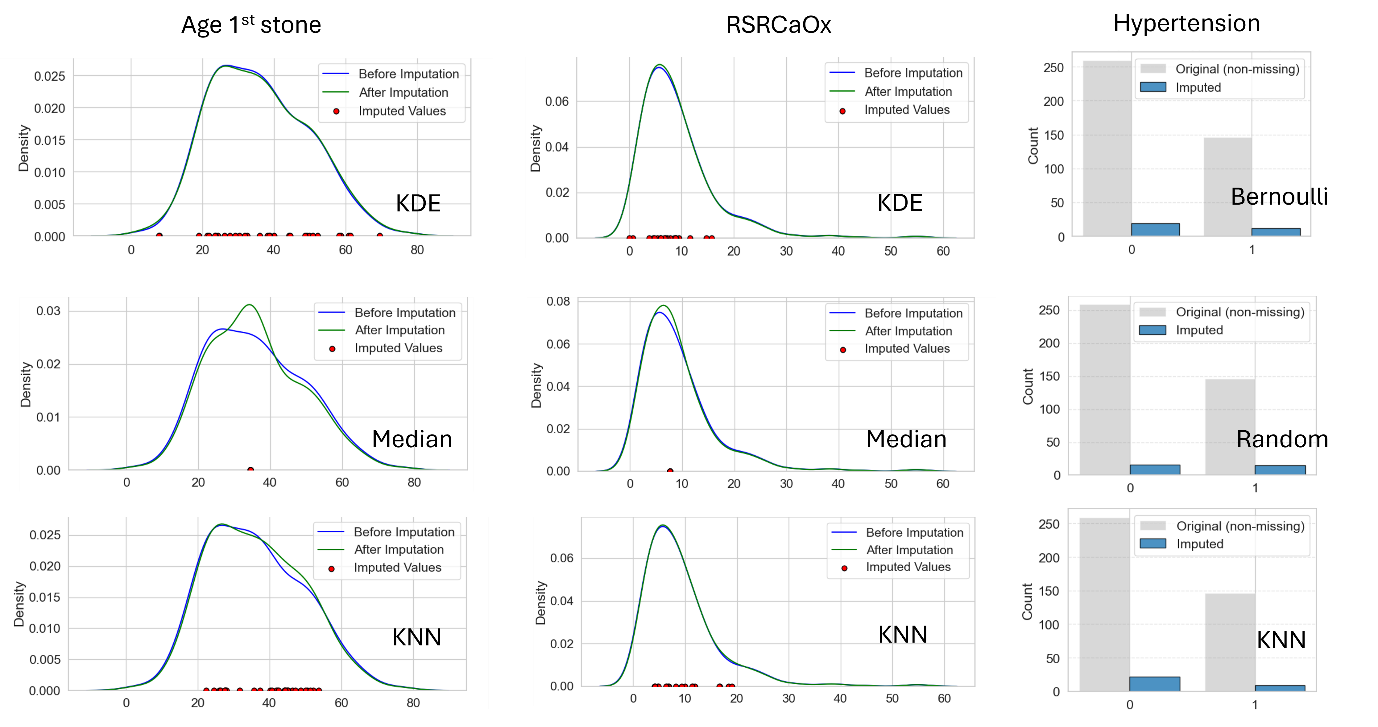


**Figure S1 - Distribution of imputed data across methods.** Distributions of age of 1^st^ stone, RSRCaOx, and hypertension before and after imputation using KDE, median, and KNN methods. Plots illustrate how each method affects data distribution.


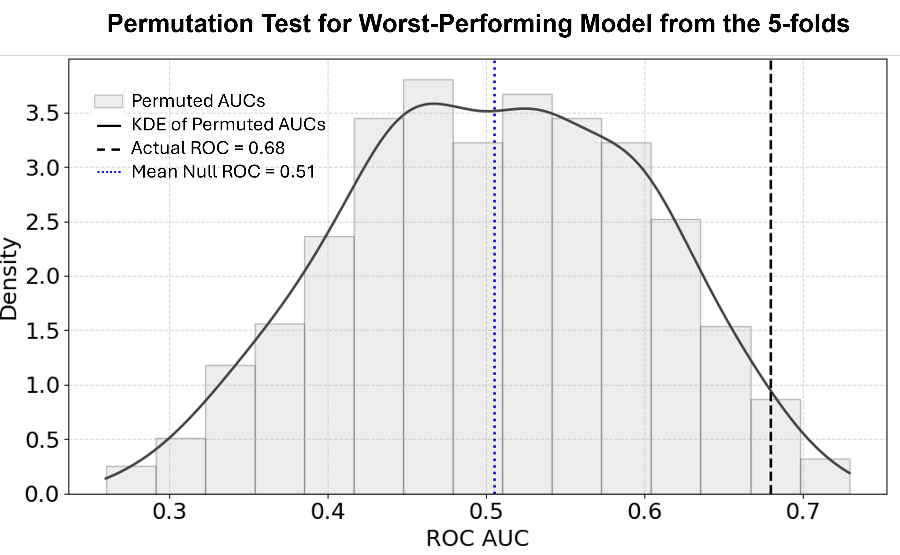


**Figure S2 -** Permutation test for the worst performing model out of the 5 folds.
